# Imepitoin for treatment of idiopathic head tremor syndrome in dogs: A randomized, blinded, placebo‐controlled study

**DOI:** 10.1111/jvim.15955

**Published:** 2020-11-07

**Authors:** Nina Schneider, Heidrun Potschka, Sven Reese, Franziska Wielaender, Andrea Fischer

**Affiliations:** ^1^ Centre for Clinical Veterinary Medicine LMU Munich Munich Germany; ^2^ Institute of Pharmacology, Toxicology, and Pharmacy, LMU Munich Munich Germany; ^3^ Department of Veterinary Sciences, Faculty of Veterinary Medicine LMU Munich Munich Germany

**Keywords:** canine, dyskinesia, epilepsy, head bobbing, medical treatment, movement disorders

## Abstract

**Background:**

Idiopathic head tremor syndrome is a paroxysmal movement disorder of unknown etiology. Spontaneous remission may occur, but owners may request treatment in severely affected dogs with continued episodes. Controlled studies of the disease are not available.

**Hypothesis/Objectives:**

A drug with gamma amino butyric acid‐ergic and anxiolytic effects will decrease head tremor episodes.

**Animals:**

Twenty‐four dogs with severe nonremitting head tremor and presumptive clinical diagnosis of idiopathic head tremor syndrome.

**Methods:**

Prospective, blinded, placebo‐controlled clinical trial to compare imepitoin with placebo in dogs with frequent episodes of idiopathic head tremor. Evaluation of efficacy used the quotient T2/T1 that represented prolongation of the head tremor‐free period compared to a 3‐month baseline. A dog was considered a responder if tremors subsided or if the head tremor‐free period was 3× longer than the longest period during baseline (T2/T1 ≥ 3). Sample size calculations considered a large effect of imepitoin on T2/T1 (Cohen's *d* = 0.8).

**Results:**

There were no responders in the placebo group (0/12). In the imepitoin group, the responder rate was 17% (2/12; *P* = .18) with T2/T1 3.8 and 4.0. Mean T2/T1 was 1.0 ± 1.4 in the imepitoin and 0.4 ± 0.4 in the placebo group (*P* = .37).

**Conclusion and Clinical Importance:**

Imepitoin did not result in a significant overall benefit. Future studies should focus on treatment of subgroups with a common pathophysiology and similar comorbidities.

**Abbreviations**
delta F: decrease in mean number of monthly HT/HB days during study phase (F2) compared to baseline (F1) (delta F(%) = (1‐F2/F1) × 100). Secondary efficacy variable (conventional outcome variable)
F1: mean number of HT/HB days per month during the baseline period (monthly HT/HB frequency [days])
F2: mean number of HT/HB days per month during the study phase
HT/HB: idiopathic head tremor/head bobbing
T1: the longest interval (days) between 2 HT/HB days during the 3 months baseline period
T2: the interval (days) between the second and the third HT/HB day during the study phase
T2/T1: quotient of T2 to T1, represents the prolongation of the head tremor free period during study phase compared to baseline. Primary efficacy variable

## 1 INTRODUCTION

Idiopathic head tremor (HT; also described as head bobbing syndrome, HB) is an idiopathic paroxysmal movement disorder in dogs, with Doberman Pinschers, Bulldogs, Boxers, and Labrador Retrievers being overrepresented among affected dogs.^1^, ^2^ Clinical appearance is characterized by sudden onset of short episodes with horizontal or vertical rhythmical tremor‐like movements of the head without loss of responsiveness.^1^, ^3^ Usually, these episodes can be interrupted by distraction of the dog (eg, by offering food).^1^, ^2^ In Doberman Pinschers, a study described a wide variation in appearance ranging from early‐onset familial to late‐onset sporadic forms.^1^ The unpredictable occurrence of HT episodes resembles the clinical course of epilepsy and paroxysmal dyskinesias.

The pathophysiologic basis of HT/HB still is unresolved, but a movement disorder with parallels to dystonia or essential tremor has been considered.^1^, ^3^, ^4^, ^5^ Stressful events appear to play an important role as provocative factors and increase the occurrence of HT/HB episodes.^1^, ^2^

Many dogs display a low frequency of episodes or even experience spontaneous remission of HB.^2^, ^3^ Yet there appears a need for effective treatment approaches in some severely affected dogs. One study reported that 17% of the owners of a cohort of dogs with HT/HB requested treatment in veterinary practice.^2^ So far, medications only have been tried under uncontrolled circumstances.^1^, ^2^

The imidazolone derivative imepitoin (Pexion, Boehringer Ingelheim) acts as a low affinity, partial agonist at the benzodiazepine recognition site of the gamma amino butyric acid A (GABA_A_) receptor and is authorized in Europe for decreasing of the frequency of generalized seizures caused by idiopathic epilepsy in dogs and anxiety and fear associated with noise phobia in dogs.^6^, ^7^, ^8^ Imepitoin is licensed for use in dogs at a dosage of 10‐30 mg/kg q12h in dogs with idiopathic epilepsy or at a dosage of 30 mg/kg q12h for treatment of noise phobia in dogs.^6^ We hypothesized that imepitoin could be beneficial in HT/HB patients because it would provide anticonvulsant and muscle relaxation properties as well as minimizing anxiety caused by stressful events as a trigger for HT/HB episodes. We aimed to evaluate the efficacy of imepitoin in dogs severely affected by idiopathic HT/HB syndrome in a prospective, randomized, placebo‐controlled, double‐blinded study.

In consideration of the latest suggestions of the International Veterinary Epilepsy Task Force on the design of future treatment studies, efficacy evaluation was based on individual outcome analysis and prolongation of the HT/HB‐free period compared to baseline.^9^

## 2 MATERIALS AND METHODS

**FIGURE 1:**
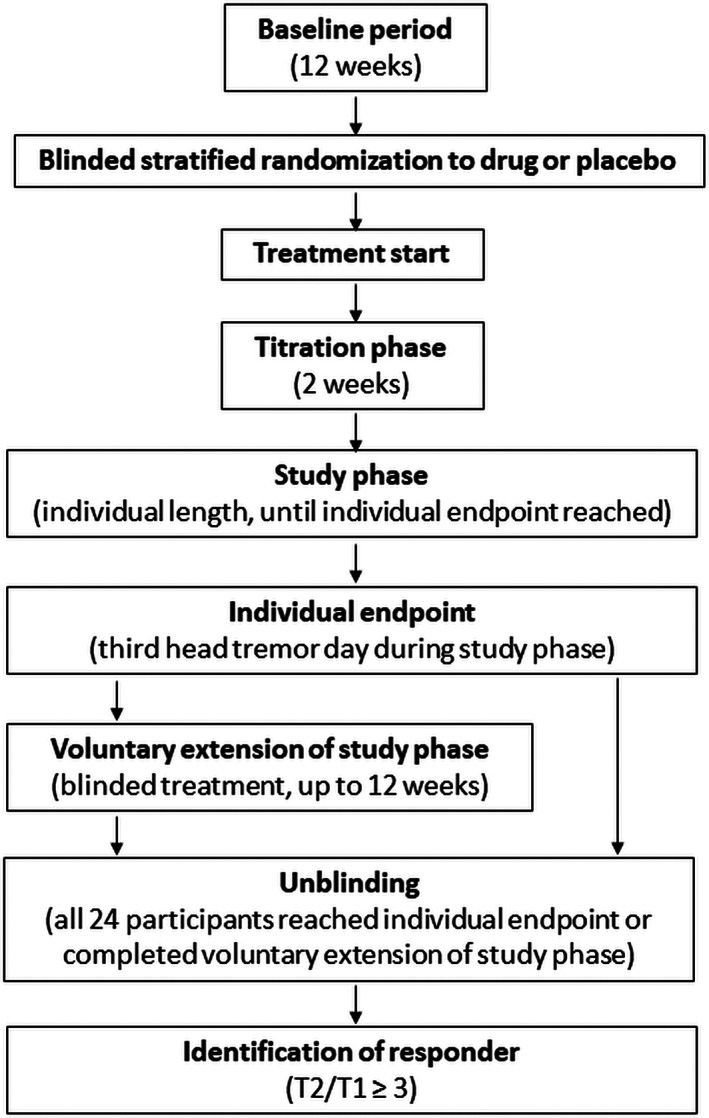
Model scheme of study. Flow chart of study. Response to treatment was evaluated based on the prolongation of the head tremor free period during study phase (T2) compared to baseline (T1). A dog was considered a responder, if tremors subsided completely or if the tremor free time was prolonged to ≥3× T1 (T2/T1 ≥ 3). T1, the longest interval (days) between 2 days with head tremor during the 3‐month baseline period; T2, the interval (days) between the second and the third day with head tremors during study phase

Our study was a prospective, randomized, double‐blinded (the owners of the dogs and the study conductors were blinded), placebo‐controlled clinical trial. Dogs were given either imepitoin or placebo tablets q12h until individual study endpoints were reached. Dogs and their owners had the option to continue treatment with either the active drug or placebo for a total treatment time of 3.5 months (14 weeks) including a 2‐week titration phase. Study conductors and participants remained blinded throughout the treatment phase (Figure 1).

### 2.1 Participants

#### 2.1.1 Recruitment

Dogs with a presumptive clinical diagnosis of HT syndrome were recruited by calls using different media and collaboration with other veterinary practices. Initially, dog owners were asked to complete an online questionnaire, including data on age, breed, and sex of the dogs, detailed description of the appearance of the HT/HB episodes and information on previous diagnostic testing, preexisting diseases, use of drugs, and any stressful events in the previous 3 months that might have triggered HT/HB (Table S1). Submission of video of the episodes in question was required for confirmation of idiopathic HT/HB.

Inclusion criteria for participants for the clinical trial were a completed questionnaire; whether or not HT/HB episodes could be interrupted; responsiveness during an HT/HB episode; submission of video of HT/HB; a history of HT/HB for at least 3 months with at least 1 HT/HB day occurring each month; documentation of HT/HB episodes in a tremor diary; and submission of the tremor diary for review by the first author of the study (N. Schneider). Dogs were excluded, if they had severe concurrent diseases such as renal, hepatic, or cardiac disease or epilepsy with convulsive seizures, were pregnant or lactating bitches. Owners of potential participants were invited to participate in a clinical, neurologic, and laboratory examination (hematology, serum biochemistry, bile acid concentration, serum concentrations of vitamins B1, B2, B6, B12). Imaging of the brain by magnetic resonance imaging and cerebrospinal fluid analysis were desirable but not required for study inclusion. Owners were offered an electroencephalographic (EEG) recording of their dogs, but EGG was not required for study inclusion. Treatment with antiseizure drugs (eg, phenobarbital) was not permitted during the 4 weeks before study initiation. Dogs were enrolled in the treatment study and blindly assigned to treatment groups, if they fulfilled the above inclusion criteria and if there were no relevant findings in the examinations.

### 2.2 Study

#### 2.2.1 Baseline

For baseline data, dogs' owners completed a tremor diary, reporting HT/HB each day during a 3‐month baseline period and any days with clusters of HT/HB. Owners were given a preprinted tremor diary on first contact for prospective data collection. Therefore, baseline data were documented in a retrospective (data before first contact) and prospective manner.

#### 2.2.2 Assignment to study groups and blinding

Participants were allocated to pairs with similar clinical characteristics following a stratified randomization procedure (Table S2). Characteristics were graded with C1 being the most and C5 being the least influencing characteristic. The major confounding factor (C1) was T1, the longest interval between 2 HT/HB episodes during baseline, followed by the factor C2 that represented the monthly HT/HB frequency. For more details, see Table S2. The company provided active drug and placebo in identical form and identical bottles for each pair of participants (12 pairs, 24 participants, labeled A and B for each pair) so that the study conductors remained blinded to the content of the bottles. Blinding was maintained until the required number of participants had completed the study and statistical analyses were completed.

#### 2.2.3 Treatment

Dogs were treated with identical tablets containing either active drug (imepitoin) or placebo q12h. Dosing was based on body weight (Table 1) aiming for an initial dose of 10 to 20 mg imepitoin/kg q12h during the first week and 20 to 30 mg/kg imepitoin q12h thereafter. Owners were instructed to give the drug at regular 12 hours intervals. Owners also were provided with clear guidance on how to handle delayed applications (Table S3).

**TABLE 1:** Dosing scheme for imepitoin 400 mg tablets and placebo

	Body weight (kg)				
	7.0‐9.9	10.0‐14.9	15.0‐20.0	20.1‐30.0	30.1‐40.0
Titration phase (days 1‐7)					
Tablets	0.25 q12h	0.5 q12h	0.5 q12h	1 q12h	1 q12h
Imepitoin total (mg)	100 q12h	200 q12h	200 q12h	400 q12h	400 q12h
Imepitoin (mg/kg)	10‐14 q12h	13‐20 q12h	10‐13 q12h	13‐20 q12h	10‐13 q12h
Titration phase (days 8‐14) and study phase					
Tablets	0.5 q12h	0.75 q12h	1 q12h	1.5 q12h	2 q12h
Imepitoin total (mg)	200 q12h	300 q12h	400 q12h	600 q12h	800 q12h
Imepitoin (mg/kg)	20‐29 q12h	20‐30 q12h	20‐27 q12h	20‐30 q12h	20‐27 q12h

Abbreviation: q12h, 2 times per day.

#### 2.2.4 Monitoring

Owners were provided with a diary to document the time of administration of tablets and to document each day with a HT/HB episode, to mark days with clusters of HT/HB and to make additional notes on the perceived severity of HT/HB. Furthermore, owners were instructed to note any stressful or other events and any co‐medication. Additionally, owners were provided with an email address and a mobile phone number to contact the study investigator at any time during the study if necessary. The dog owners were contacted by the first author (N. Schneider) of the study weekly during the 2‐week titration phase, and then every 2 or 4 weeks dependent on individual frequency of HT/HB episodes. Contact with the owners occurred via email or phone call by the first author (N. Schneider) to evaluate the occurrence of HT/HB and any adverse events.

#### 2.2.5 Treatment phase

The treatment phase consisted of a 2‐week titration phase and a subsequent study phase. The individual study endpoint was defined as the third HT/HB day during the study phase (see Figure 1). Dog owners were offered an opportunity to withdraw from the study when this event occurred.

#### 2.2.6 Assessment of efficacy

Data from the baseline period (3 months) and study phase were collected to calculate the variables T1, the longest interval (days) between 2 HT/HB days during the 3‐month baseline period; T2, the interval (days) between the second and the third HT/HB day during the study phase; and the number of cluster days (days with occurrence of ≥2 HT/HB episodes within 24 hours). Response to treatment was evaluated based on prolongation of the HT/HB‐free period. The quotient of T2/T1 was calculated as the primary efficacy variable. A dog was considered a responder if tremors subsided completely or if the tremor‐free time was prolonged to ≥3 × T1 (T2/T1 ≥ 3). A dog was considered a partial responder if the tremor‐free time was prolonged by at least 50% (T2/T1 ≥ 1.5) or if there were fewer cluster days.

#### 2.2.7 Voluntary extension

Dog owners were offered the opportunity to continue the blinded treatment voluntarily up to a total treatment time of 14 weeks or longer until the dispensed study compound was depleted.

For all dogs treated for ≥6 weeks (study phase and voluntary extension), additional variables were calculated: F1, mean number of HT/HB days per month during the baseline period (monthly HT/HB frequency [days]) and F2, mean number of HT/HB days per month during the study phase. Delta F was assessed as a secondary efficacy variable (conventional outcome variable). Delta F was defined as %‐decrease in mean number of monthly HT/HB days during study phase (F2) compared to baseline (F1; delta F [%] = [1‐F2/F1] × 100). Positive results indicated a decrease in HT/HB days and negative results indicated an increase in HT/HB days. Thereby, responders were defined by a ≥50% decrease in HT/HB day frequency.

#### 2.2.8 Monitoring for adverse events

Any abnormal health observation that was unfavorable, unintended, and occurred after enrollment, regardless of whether or not it was considered a treatment‐related event, was reported to the investigator and a standard adverse event reporting form was sent to the company. Details of the adverse event reports included the following: a description of the adverse event; onset of signs; duration of the adverse event; severity of the adverse events (mild/moderate/severe); treatment of the adverse event; outcome of the individual study participant; withdrawal of the study participant because of an adverse event; outcome of rechallenge after withdrawal; and assessment of the potential association between treatment and adverse event.

#### 2.2.9 Management of adverse events

Both the owners of the dogs and the study conductors remained blinded during the management of adverse events. In case of unacceptable adverse events at 20 to 30 mg/kg imepitoin q12h or an equivalent number of placebo tablets imepitoin was decreased to 10 to 20 mg/kg q12h or an equivalent number of placebo tablets in agreement with the owner. In case of unacceptable adverse events at 10 to 20 mg/kg q12h, the dog was excluded from the study. The severity of the adverse event was evaluated by the owner and investigator with respect to impairment of the patient's general condition and influence on the daily routine of the dog and owner.

### 2.3 Statistical analysis

The study was designed to detect a large effect (Cohen *d* = 0.8) of the active compound imepitoin on T2/T1. A 1‐tailed paired *t* test and Wilcoxon test (that was able to evaluate a positive effect) indicated a sample size of at least 12 dogs in each group as necessary to detect a large effect (power 80%, first‐degree error, 0.05; G*Power, 3.1^10^). Comparison of baseline characteristics between study groups (imepitoin, placebo) was done using the chi‐square test, binomial test, and McNemar test (SPSS 25.0 IBM and BIAS for Windows 11.01; epsilon Frankfurt). A comparison of T2/T1 between study groups was done using a 1‐tailed paired *t* test. Delta F between groups was assessed using the Wilcoxon test. Significance level was *P* < .05.

## 3 RESULTS

In total, 427 owners of dogs with HT/HB completed the online questionnaire. Twenty‐five dogs with a presumptive clinical diagnosis of idiopathic HT/HB syndrome fulfilled the inclusion criteria. The main reason for exclusion was recent onset of HT/HB which subsequently subsided. One dog failed to enter the study phase because of adverse events during the titration phase (day 9). Thus, 24 dogs were included in the final data set for evaluation of efficacy.

#### 3.1.1. Study population (n = 24)

**FIGURE 2:**
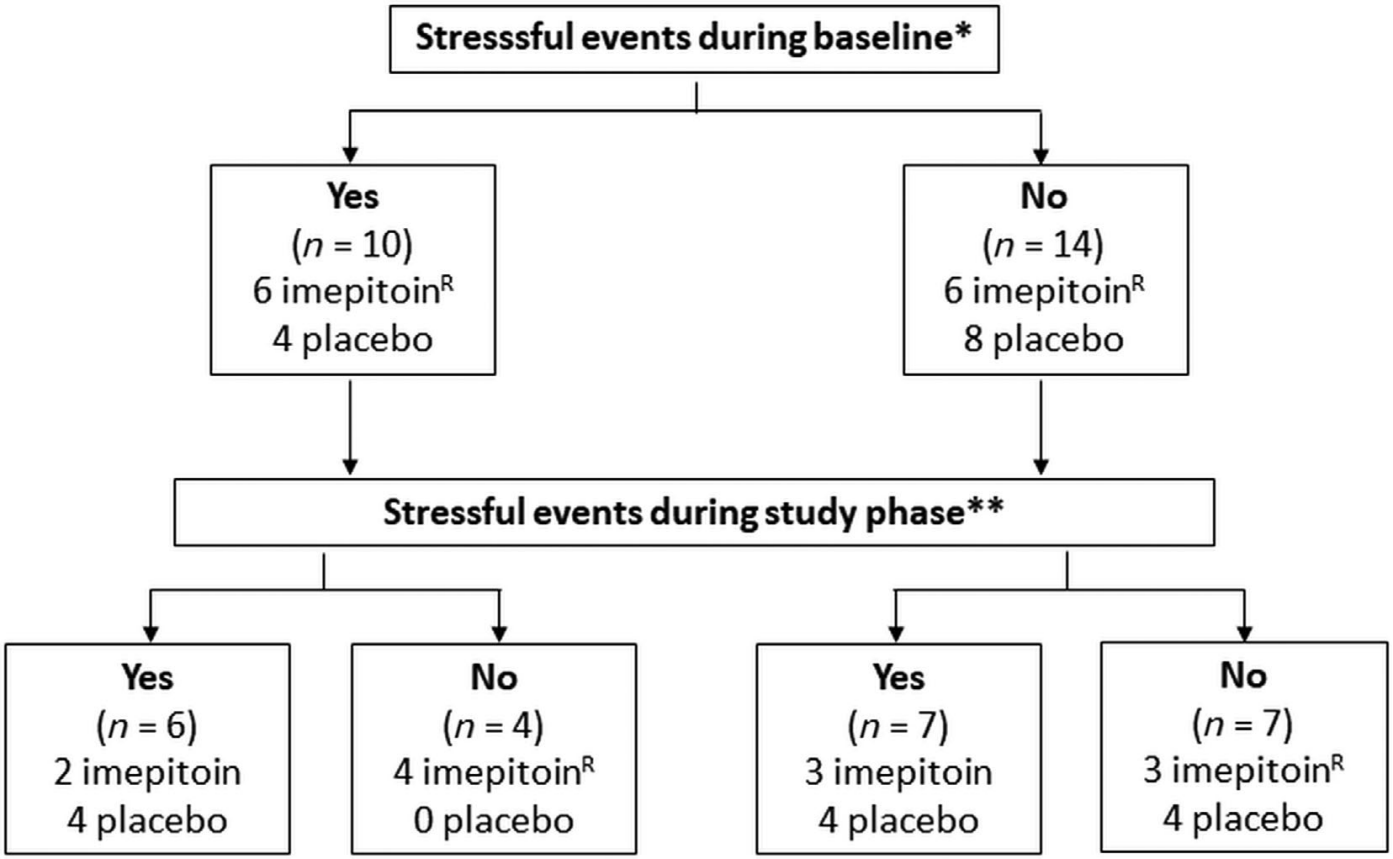
Reports of stressful events in study participants (n = 24). There was no difference in reports of stressful events in dogs assigned to imepitoin or placebo during baseline. During treatment, one responder (R) experienced no stressful events. The other responder (R) experienced stressful events during baseline but not during treatment. *reports of stressful events during baseline: Change of owner, pregnancy or heat in bitches, conflicts with another dog in the same household, death of another dog in the same household, medicaments (meloxicam), thunderstorm, dog training, house moving. **Reports of stressful events during study phase: heat in bitches, vomitus, hot temperatures, holidays, dog training, fireworks, loud noises, stress of owner, fever, visitors, absence of the owner, building work, veterinary consult

The mean age of onset of HT/HB was 2.4 years. Dogs had shown frequent HT/HB episodes for 1.7 years (mean) at the time of inclusion. Owners of 41% (10/24; imepitoin, n = 6; placebo, n = 4) of the dogs reported that stressful events occurred during the previous 3 months that might have triggered at least 1 episode of HT/HB (see Figure 2). A previous therapeutic trial was reported in 40% of dogs, mostly using various preparations of vitamin B supplements. One dog had been treated with phenobarbital. Neither treatment had an impact on the occurrence of HT/HB. Baseline data for dogs assigned to the imepitoin or placebo group are presented in Table 2. Besides mean T1 (imepitoin, 27.6 days; placebo, 16.8 days; *P* = .004), no differences in patient or disease characteristics were observed between the 2 groups (Table 2).

**TABLE 2:** Baseline data for evaluation of efficacy (n = 24)

	Total, n = 24	Imepitoin, n = 12	Placebo, n = 12	*P* value
Breeds				
Predisposed breeds (total)	20/24	11/12	9/12	.82
Bulldogs	14/24	8/12	6/12	.79
Doberman Pinscher	5/24	2/12	3/12	1.00
Boxer	1/24	1/12	—	—
Nonpredisposed breeds	4/24	1/12	3/12	.62
Body weight (mean)	27 kg (8‐42 kg)	26 kg (8‐42 kg)	30 kg (12‐42 kg)	.27
Sex	15 males	6 males	9 males	.61
	9 females	6 females	3 females	.51
Age of onset (mean)	2.4 y (5 mo‐9 y)	2.4 y (6 mo‐5 y)	2.5 y (5 mo‐9 y)	.48
Age at inclusion (mean)	4.2 y (8 mo‐9 y)	3.9 y (2.5‐7 y)	4.4 y (8 mo‐9 y)	1.00
F1 (mean)	7 (1.2‐28)	5.4 (1.2‐23.7)	8.6 (1.6‐28)	.21
T1 (mean)	22.2 d (1‐52 d)	27.6 d (5‐52 d)	16.8 d (1‐35 d)	.004
Cluster days	11/24	6/12	5/12	1.00
Stressful events	10/24	6/12	4/12	.75

*Note*: Baseline data of dogs that were included in the final data analysis for efficacy.

Abbreviations: F1, mean number of head tremor days per month during the baseline period (monthly head tremor frequency) 2 times a day; T1, the longest interval between 2 head tremor days during the 3 months baseline period.

### 3.2 Assessment of efficacy

#### 3.2.1 Study phase

All dogs that entered the study phase reached their individual study endpoint. The T2/T1 ratio was higher in the imepitoin group (1.0 ± 1.4) than in the placebo group (0.4 ± 0.4), but the difference failed to reach statistical significance (*P* = .37). In the imepitoin group, 2 dogs were classified as responders (T2/T1 > 3; dogs 7, 12) and 1 dog was classified as a partial responder based on T2/T1 (T2/T1 > 1.5; dog 14). No responder or partial responder was identified in the placebo group based on T2/T1 (*P* = .18; Table 3). The responder rate (partial responders not included) amounted to 17% in the imepitoin group and 0% in the placebo group, based on T2/T1. For detailed study patient data see Table 3 and Table S4.

**TABLE 3:** Evaluation of treatment response (n = 24)

	Imepitoin, n = 12	Placebo, n = 12	*P* value
Mean T1 (baseline) (±SD)	27.6 (±13.7)	16.8 (±10.8)	.004
Mean T2 (study phase) (±SD)	24.6 (±32)	4.2 (±3.5)	.01
Mean T2/T1 (± SD)	1.0 (±1.4)	0.4 (±0.4)	.37
Responder (T2/T1 ≥ 3)	2	0	
Partial responder (T2/T1 > 1,5 and < 3)	1	0	
Nonresponder (T2/T1 < 1,5)	9	12	
Responder rate	17%	0%	.18

*Note*: Response to treatment was evaluated based on the prolongation of the head tremor‐free period, wherefore the quotient T2/T1 was calculated as the primary efficacy variable.

Abbreviations: T1, the longest interval (days) between 2 head tremor days during the 3‐month baseline period; T2: interval (days) between the second and the third head tremor day during study phase after completion of the titration phase.

#### 3.2.2 Cluster

A nonsignificant decrease in HT/HB cluster days occurred in the imepitoin group (*P* = .64; Table S5). Three additional dogs from the imepitoin group (responder and partial responder based on T2/T1 excluded) and 3 dogs from the placebo group were partial responders, based on improvement in the number of cluster days.

#### 3.2.3 Voluntary extension

Six dogs of the imepitoin group and 9 dogs of the placebo group continued blinded treatment voluntarily after their individual study endpoints were reached. No difference was found in the total treatment duration between the imepitoin and the placebo groups (Table 4).

**TABLE 4:** Total treatment duration

Total treatment duration	Imepitoin, n = 12		Placebo, n = 12	
(in days)	Responder	Nonresponder	Responder	Nonresponder
0‐30	—	2	—	5
>30‐60	1	1	—	2
>60‐90	—	4	—	2
>90	2^a^	2	—	3
Mean ± SD	67.8 ± 36.0		70.4 ± 60.9	

*Notes*: Number of dogs and their total treatment duration. The total treatment duration is defined as the study phase and the voluntary extension if dogs' owners decided to continue treatment after the third HT/HB. There was no difference in mean treatment duration between the imepitoin and the placebo group.

^a^ One dog was a responder, the other dog was a partial responder.

#### 3.2.4 Decrease in monthly HT/HB frequency (delta F in %)

Fourteen dogs (7 pairs) remained in the study for ≥6 weeks. Additional evaluation for responders was based on delta F for these dogs. Mean delta F was in the same range in the imepitoin group (delta F = 36%) and in the placebo group (delta F = 30%; *P* = .5). The same dogs from the imepitoin group that were classified as responders (dogs 7, 12) or partial responder (dog 14) based on T2/T1 data were classified as responders by delta F (dogs 7, 12, 14). However, 2 more dogs were classified as responders by delta F in the placebo group (dogs 11, 13). The difference in responder rates between imepitoin and placebo group failed to reach statistical significance (*P* = .96; Table 5). For detailed study patient data see Table S6.

**TABLE 5:** Reduction in monthly head tremor days frequency (delta F) for dogs treated ≥6 weeks (n = 14)

	Imepitoin, n = 7	Placebo, n = 7	*P* value
Mean F1 ± SD	4.6 ± 2.9	5.0 ± 3.6	1.00
Mean F2 ± SD	2.1 ± 1.7	2.7 ± 1.4	.49
Mean delta F (range)	36% (−68% to 100%)	30% (−51% to 78%)	.49
Delta F ≥ 50%	3	2	
Delta F ≥ 0% and <50%	3	3	
Delta F < 0%	1	2	
Responder rate	42%	29%	.97

*Notes*: Reduction in monthly head tremor days frequency (delta F) was defined as %‐decrease in mean number of monthly head tremor days during study phase compared to baseline. Positive results indicated a decrease in head tremor days, negative results indicated an increase in head tremor days. Responders were defined by ≥50% decrease in head tremor day frequency. Delta F was evaluated for all dogs treated ≥6 weeks (n = 14).

Abbreviations: F1, mean number of head tremor days per month during the baseline period (monthly head tremor frequency); F2, mean number of head tremor days per month during the study phase.

#### 3.2.5 Characterization of responders based on T2/T1

There was no obvious difference in patient and disease characteristics between responders and nonresponders (Table 6). Responders were bulldogs that showed a HT/HB with a horizontal direction of head movement. However, bulldogs and dogs with horizontal HT/HB generally were overrepresented in the study (imepitoin, n = 9; placebo, n = 11). The T2 was 3.8 and 4.0 times longer than T1 for the 2 responders and 1.6 times longer than T1 for the partial responder (Table 6).

**TABLE 6:** Characteristics of responders compared to nonresponders from the imepitoin group

	Responder I	Responder II	Partial responder	Nonresponders (Imepitoin)
	Dog No 7	Dog No 12	Dog No 14	9 dogs^a^
Signalment				
Breed	French Bulldog	English Bulldog	Continental Bulldog	—
Sex (neutered/intact)	Male (intact)	Female (intact)	Male (intact)	—
Weight in kg	13	23	38	26
Age in years	3.9	2.9	5.3	3.8
Characteristics of HT/HB				
Age of onset (years)	3.5	1.9	4.9	2
Time since onset at time of inclusion (years)	0.4	1.0	0.5	1.8
Direction of HT/HB	Horizontal	Horizontal	Horizontal	—
Duration of HT/HB	1 min	>5 min	>5 min	—
Baseline data				
T1 in days	29	14	28	29
F1 in days per month	6.5	3.1	10.0	5.1
Study phase data				
T2 in days	109	56	46	9.3
F2 in days per month	0.4	0	2.9	4.9
T2/T1	3.8	4.0	1.6	0.3
Treatment duration in days (after titration phase)	126	57	117	57

*Note*: Baseline data (patient and disease characteristics) and study phase data of responders and partial responders in comparison with nonresponders of the imepitoin group (n = 9); evaluation based on T2/T1 as individual outcome variable (prolongation of HT/HB free period).

Abbreviations: F1, mean number of head tremor days per month during the baseline period (monthly head tremor frequency); F2, mean number of head tremor days per month during the study phase; HT/HB, head tremor/head bobbing; T1, the longest interval between 2 head tremor days during the 3‐month baseline period; T2: interval between the second and the third head tremor day during study phase after completion of the titration phase.

^a^ Data are shown as the mean of all 9 dogs from the imepitoin group classified as nonresponders.

#### 3.2.6 Stressful events

Forty‐one percent (10/24) of dog owners reported a stressful event that might have triggered at least 1 HT/HB episode during baseline. In 4 of these 10 dogs, no triggers were identified during the study phase. One responder (dog 12) and the partial responder (dog 14) belonged to this group of dogs. For the other responder (dog 7), no stressful events were identified as a potential trigger during baseline nor during the study phase. For details, see Figure 2.

#### 3.2.7 Adverse events

All 25 randomized dogs were included in the final data analysis for adverse events. Five dogs that were assigned to imepitoin (5/13; 38.5%) and 3 dogs of the placebo group (3/12; 25%) experienced adverse events (*P* < .05). Adverse events were mild or moderate in severity and severe adverse events were not reported (Table 7).

**TABLE 7:** Adverse events (n = 25)

	Imepitoin, n = 13	Placebo, n = 12
Number of adverse events	5	6
Number of dogs with adverse events	5	3
Number of excluded dogs because of adverse events	1	0
Number of dogs with dose reductions	3	2
Organ system affected and number of affected dogs		
Central nervous system (ataxia)	1	1
Gastrointestinal (vomiting, diarrhea)	1	2
Skin (pruritus)	1	0
Polyphagia	1	0
Worsening of HT/HB	0	2
Noise sensitivity	0	1
Restlessness	1	0

*Notes*: One dog of the imepitoin group failed to enter the study phase because of ataxia during the titration phase. This dog is included in the analysis of adverse events but not in the evaluation of efficacy.

Abbreviation: HT/HB, head tremor/head bobbing.

#### 3.2.8 Imepitoin group

One dog failed to enter the study phase because of moderate ataxia that appeared during titration when the initial dosage was increased to 20 to 30 mg/kg imepitoin q12h (day 9 of titration phase). Data from this dog were not considered in the final data analysis of efficacy. The dog recovered completely after discontinuing the tablets but experienced the same adverse event during re‐challenge using a lower dosage of 10 to 20 mg/kg. In 2 dogs, the dosage of 20 to 30 mg/kg q12h was decreased to 10 to 20 mg/kg because of polyphagia and restlessness. Full recovery occurred after dose decrease and the lower dose was continued for the remainder of the study. In 2 other dogs, clinical signs were very mild (transient pruritus) or appeared as a single event (vomiting) and the dose was not changed. None of these dogs experienced additional adverse events during the study phase.

#### 3.2.9 Placebo group

The owners of 3 dogs reported adverse events with placebo tablets at a dose that was equivalent to the presumptive dosage of 20 to 30 mg/kg imepitoin q12h. Intermittent worsening of HT (defined as an increase in intensity and duration of HT) was reported in 2 dogs and resolved spontaneously. One of these dogs also experienced vomiting, diarrhea and ataxia at different times during treatment. Vomiting and diarrhea resolved spontaneously, and ataxia resolved after placebo dose decrease. The third dog experienced mild noise sensitivity that persisted even with a decreased dose of placebo (Table 7).

## 4 DISCUSSION

Our study was designed to evaluate the short‐term efficacy of the low‐affinity, partial benzodiazepine receptor agonist imepitoin in dogs with idiopathic HT/HB syndrome in a prospective, randomized placebo‐controlled double‐blinded treatment trial.

Twenty‐four dogs completed the study and were included in the final analysis. Responder rate was 17% in the imepitoin group and 0% in the placebo group. There was 1 additional partial responder in the imepitoin group. Differences between the treatment and placebo groups were too small to reach statistical significance. The results indicate however that a few dogs with a presumptive clinical diagnosis of idiopathic HT/HB syndrome may respond to imepitoin with a 3× increase in tremor‐free time or even resolution of HT/HB.

The number of dogs with reports of adverse events was higher than the number that benefited from treatment, but adverse events were mild or transient and resolved spontaneously or with a decreased dose of imepitoin in 4 dogs. One dog however failed to enter the study phase because of ataxia during the titration phase. It could be debated whether this dog should be included in the final data analysis for efficacy. Inclusion of this dog would follow an intention‐to‐treat analysis and would decrease the responder rate from 17% to 15% (2/13). However, our aim was to focus on investigation of efficacy of imepitoin for HT/HB and therefore we chose to analyze the data sets of patients that started the study phase (n = 24) rather than an intention‐to‐treat approach with missing outcome data.^11^, ^12^ This dog however was included in the data set for analysis for adverse events.

It is interesting that adverse events also were reported in the placebo group. Worsening of HT/HB in 2 dogs could represent lack of efficacy of placebo or just natural fluctuation of the disease. Events reported during a study are not necessarily directly linked with the exposure. Vomiting and diarrhea might have arisen from incidental gastroenteritis. In the human medical literature, the occurrence of adverse events in placebo‐treated groups is described as nocebo effect.^13^, ^14^ Although no veterinary studies address whether the nocebo effect exists in animals, a recent placebo‐controlled clinical trial with imepitoin in dogs described adverse events in the placebo group, including ataxia.^8^

Imepitoin was used in our clinical trial for its good tolerability and modulation of GABAergic activity, which may play a role in epilepsy and movement disorders.^15^, ^16^, ^17^, ^18^, ^19^ Imepitoin also may have anxiolytic effects and therefore could modulate the response to stressful events.^7^, ^20^ Imepitoin's GABAergic activity is based on its ability to bind the same recognition site of the GABA_A_ receptor as benzodiazepines, but it acts as a low affinity and partial agonist.^6^, ^21^

Etiology of the HT syndrome is still a matter of debate, and idiopathic HT/HB may be the common clinical presentation of diverse etiologies. Although in the past an epileptic syndrome was considered unlikely because of a lack of autonomic signs and impact on responsiveness and failure to identify epileptiform activity in anesthetized dogs, an epileptic origin again has been considered based on suggestive ictal EEG recordings.^1^, ^3^, ^4^, ^5^, ^22^ On the other hand, HT also is considered a movement disorder with parallels to dystonia or essential tremor.^1^, ^3^, ^4^, ^5^ Essential tremor is a syndrome in humans particularly characterized by tremor of the upper limbs.^23^, ^24^ Although additional tremors also may occur in the head, a recent consensus statement on the classification of tremors in human medicine termed isolated focal tremors as exclusion criteria for essential tremor.^23^ Essential tremor is thought to represent a syndrome with a variety of etiologies.^23^, ^24^ A dysfunction of the GABAergic system or an abnormal ratio between GABA and glutamate has been hypothesized as the underlying cause leading to an imbalance between the excitatory and inhibitory systems.^25^, ^26^ Some GABA receptor agonists such as clonazepam are recommended as a second‐line treatment option, whereas propranolol and primidone are still the first choices for medical management of essential tremor.^27^, ^28^ Dystonia is defined as “involuntary sustained or intermittent muscle contractions causing abnormal, often repetitive, movements, postures, or both.”^17^ Dystonia shows a wide spectrum of clinical appearance and also can lead to tremor‐like symptoms. Coexistence with other movement disorders has been described.^17^ A tremor is defined as “involuntary, rhythmic, oscillatory movement of a body part.”^5^ Although, by definition, a tremor can be separated from dystonia because of its non‐rhythmic occurrence, isolated tremors have been described as a common feature in humans with cervical dystonia.^17^, ^29^ Thus, idiopathic HT/HB was suggested to represent a manifestation of cervical dystonia in dogs.^1^, ^30^ The pathophysiology of dystonia is not completely understood. One hypothesis is that dystonia is caused by a loss of inhibitory control of motor function because of GABA disinhibition.^15^, ^16^, ^17^ Drugs that enhance the inhibitory effect of GABA, such as benzodiazepines or other GABA receptor agonists, seem to be effective in some patients with dystonia based on small clinical trials or case reports.^17^, ^31^ However, the evidence is low and botulinum toxin is the only proven effective treatment for dystonia.^32^ In some forms of dystonia in humans, an abnormal density of GABA_A_‐receptors has been found in the cerebellum and forebrain, which supports the hypothesis of a GABAergic mechanism.^15^, ^16^ A further characteristic shared by dystonia in humans and HT in dogs is consistent ability to interrupt the episodes. This finding is reminiscent of the geste antagonist or sensory tricks that consistently stop dystonic episodes in humans.^33^ Furthermore, comorbid anxiety and stress play important roles as provocative factors in dystonia and HT in dogs.^1^, ^17^, ^34^ Anxiety is associated with hyperexcitability, and enhancing inhibition with GABAergic drugs proved to be beneficial in humans and dogs with anxious conditions.^8^, ^35^ Lastly, it is also possible that HT represents the manifestation of different pathophysiologic entities sharing the same clinical features.

Review of patient and disease characteristics of responders and nonresponders failed to show a difference (Table 6). We did not identify any predictive markers of response to imepitoin. Stressful events were reported as a suspected trigger for HT/HB episodes in 41% of dogs during baseline and 54% of dogs during the study phase. In no responder or partial responder dogs, owners reported stressful events during the study phase but in dog 12 (responder) and dog 14 (partial responder), stressful events appeared to be triggers of HT/HB during baseline. It is possible that the absence of stressful events during the study phase in dogs 12 and 14 had a positive effect on outcome in these 2 dogs. It is also possible that owners paid less attention to stressful events because of the absence of HT/HB episodes. In contrast, in other dogs, the absence of stressful events during study phase did not influence the outcome (nonresponders). It is of interest that in 50% of the 14 dogs, stressful events were not reported during baseline but were identified during the study phase. Owners might have paid more attention to triggers during study phase or stressful events may not have been reported during baseline because the owners saw no association between HT/HB and stressful events. In a study about precipitating factors in epileptic dogs, only 19% experienced an epileptic seizure immediately after exposure to a precipitating factor whereas in the other dogs seizures occurred within 24 hours or even later after a precipitating factor had occurred.^36^ Types of stressful events appeared to be very variable, and it is also possible that the change in reports during the study phase was caused by the coincidental appearance of stressful events. Thus, it remains unclear whether dogs with reported triggers of HT/HB are more likely to respond to treatment with imepitoin.

In accordance with the paroxysmal appearance of HT/HB in the HT syndrome, we used a protocol that already has been successfully applied for assessment of the response to treatment modification in dogs with idiopathic epilepsy.^37^ The study protocol was designed to investigate early efficacy and to maximize owner compliance. Conventional study designs in epilepsy studies can raise important ethical issues because patients must maintain treatment for several months to include them in the final data analysis, even if the treatment is inadequate.^9^ In our study, dog owners were permitted to exit the study when a lack of efficacy was indicated by T2/T1. This approach apparently increased owner compliance and decreased placebo responses, which can originate from a lack of recording of events by owners during prolonged treatment.

Efficacy was assessed based on prolongation of the HT/HB‐free period compared to baseline. A dog was considered a responder if tremors subsided completely or if the tremor‐free time was prolonged to ≥3× the longest interval during baseline. The study endpoint was defined as the third HT/HB day during the study phase after a 2‐week titration phase. This endpoint is in concordance with recent International League Against Epilepsy recommendations in which seizure freedom was defined as “a phase of at least 3 times the duration of their longest pre‐intervention interseizure interval in the preceding 12 months or during 12 months, whichever is longer.”^38^ It also has been suggested to use this approach in epilepsy studies in dogs.^9^ However, it has not been specified if the first, second, or other interseizure interval during the study phase should be used for evaluation. In our study, the interval between the second and third HT/HB episode after titration (T2) was used for calculation of the efficacy variable T2/T1. Additional studies are necessary to evaluate whether this interval is representative when compared to other intervals. The use of an early interval is suitable to assess short term outcomes but not later disease‐modifying effects.

Efficacy additionally was evaluated based on the decrease in monthly HT/HB frequency in 14 dogs that participated for ≥6 weeks in the study phase and during voluntary extension of the blinded treatment. In this group, dogs were classified as responders if they experienced ≥50% decrease in monthly HT/HB day frequency compared to baseline. A ≥50% reduction in seizure frequency compared to baseline is a common variable in conventional epilepsy study protocols to define responders to a certain treatment in veterinary and human medicine.^9^, ^39^, ^40^, ^41^ The same dogs from the imepitoin group were detected as responders by both evaluation concepts, respectively. However, 2 additional dogs were defined as responders in the placebo group. Thus, the evaluation system based on T2/T1 appeared to be associated with a lower placebo rate.

The main limitation of our study was that it was designed to show a large effect on T2/T1, and the number of study patients might be too small to evaluate characteristics for subgroups that could be more responsive to treatment. It is also possible that some subtype of the disorder was underrepresented in our study and thus efficacy was underestimated. Comparison of baseline data showed that mean T1 (imepitoin, 27.6 days; placebo, 16.8 days) was significantly longer and mean F1 was slightly smaller in the imepitoin group, implying that dogs from the imepitoin group were less severely affected than dogs of the placebo group. Although this may be considered a limitation of the study, the difference was compensated by the study design, which used an individual efficacy variable (T2/T1). Assignment to study groups was based on allocation of participants to pairs with similar clinical characteristics. However, the major confounding factor (C1) was T1, the longest interval between 2 HT/HB episodes during baseline, followed by the factor C2, which represented the monthly HT/HB frequency. Both factors are consistent with disease severity. In our allocation of pairs, severity and frequency of stressful events as triggers of HT/HB were not considered as independent confounding factors in the matching process.

We were unable to obtain any ictal EEG recordings from study participants. Thus, we cannot exclude the possibility of epileptiform activity in responders. The HT/HB calendar data were based on owners' documentation and compliance, and dogs were not observed 24 hours every day. Therefore, it is possible that some HT/HB episodes either were missed or were not recorded by the owners in dogs that were classified as responders. The suspected triggers for HT/HB were based on subjective assessment by the owners. Reported stressful events might have had no impact on HT/HB or relevant stressful events could have been missed by the owner.

## 5 CONCLUSION

Imepitoin did not have a significant overall benefit in our study. Nevertheless, 2 dogs with severe nonremitting HT showed improvement with imepitoin. Future studies should focus on treatment of subgroups with a common pathophysiology and similar comorbidities. More research on the underlying pathophysiology of HT/HB is required to understand this syndrome.

## CONFLICT OF INTEREST DECLARATION

Authors declare no conflict of interest. The study was a blinded, randomized placebo‐controlled study. All data were evaluated and interpreted independently from the sponsor.

## OFF‐LABEL ANTIMICROBIAL DECLARATION

Authors declare no off‐label use of antimicrobials.

## INSTITUTIONAL ANIMAL CARE AND USE COMMITTEE (IACUC) OR OTHER APPROVAL DECLARATION

Approved by the Government of Upper Bavaria (AZ 55.1‐1‐55‐2532‐227‐2015). All dogs were privately owned dogs and lived with their owners during the study. Owner consent was obtained.

## HUMAN ETHICS APPROVAL DECLARATION

Authors declare human ethics approval was not needed for this study.

## Supporting information


**Table S1** Online questionnaire ‐ Clinical evaluation of imepitoin in dogs with idiopathic episodic head tremor (“head bobbing”) (format: PDF)

**Table S2** Patient and disease characteristics for design of matched pairs (format: PDF)

**Table S3** Owner instruction for application of tablets and delayed application (format: PDF)

**Table S4** Single patient data: T1, T2, and T2/T1 in imepitoin and placebo group (format: PDF)

**Table S5** Trend of cluster days during study phase compared to baseline (format: PDF)

**Table S6** Single patient data: F1, F2 and delta F in imepitoin and placebo group (only for matched pairs treated for>6 weeks with active drug or placebo) (format: PDF)

**Table S7** Different T2/T1 quotients for study patients with voluntary extension based on different T2 (format: PDF)
