# Supplementary material for: Imepitoin for treatment of idiopathic head tremor syndrome in dogs: A randomized, blinded, placebo‐controlled study
Source: J Vet Intern Med. 2020 Nov 7;34(6):2571–81. doi: 10.1111/jvim.15955 (PMC7694850; doi:10.1111/jvim.15955)
Supplement: Supplementary file 1 — Table S1 Online questionnaire ‐ Clinical evaluation of imepitoin in dogs with idiopathic episodic head tremor (“head bobbing”) (format: PDF) [file JVIM-34-2571-s001.pdf]

**Table S1: Online questionnaire - Clinical evaluation of imepitoin in dogs with idiopathic episodic head tremor (“head bobbing”)**

| | |
|---|---|
| Date | |
| Owner’s name | |
| Owner’s adress | |
| E-mail | |
| Telephone number | |
| | |
| Dog’s name | |
| Breed | |
| Gender | □ male □ female |
| Castrated yes/no | □ yes □no |
| Body weight | |
| | |
| Age of first head bobbing | |
| Reasons or circumstances that might have triggered the appearance of head bobbing in the last 3 months | |
| Is the tremor interruptible? Yes/no | □ yes □no |
| How can you interrupt the tremor (goody, talking to the dog, touching the dog, …) | |
| Is the dog conscious? Yes/no | □ yes □no |
| Tremor type: Is the dog’s head shaking up and down (yes-yes-tremor) or from side to side (no-no-tremor)? | □ yes-yes-tremor<br>□no-no-tremor |
| Duration of one tremor episode in general | □ some seconds □ about 1 minute □ 3-5 minutes □more than 5 minutes □ I don’t know |
| When have your dog showed head bobbing the last time? | |
| On how many days have your dog shown head bobbing episodes during the last 3 months | • last month:<br>• 1 month ago:<br>• 2 months ago: |
| In the last 3 months: What has been the **shortes** time interval between two days with head bobbing? | □ 1-3 days<br>□ 4-7 days<br>□ 8-14 days<br>□ 14-21 days<br>□ 22-31 days<br>□ more<br>□ I don’t know |
| In the last 3 months: What has been the **longest** time interval between two days with head bobbing? | □ less than 7 days<br>□ 8-14 days<br>□ 14-21 days<br>□ 22-31 days<br>□ more<br>□ I don’t know |
| In the last year: What has been the time interval between two days with head bobbing **in general**? | □ less than 7 days<br>□ 1-2 weeks<br>□ 2-4 weeks |

| | |
|---|---|
| | ☐ 1 month<br>☐ 2 months<br>☐ 3-4 months<br>☐ 5-6 months<br>☐ more<br>☐ I don't know<br>☐ my dog shows the head bobbing syndrome for less than 3 months |
| In the last 3 months: What has been the **longest** time interval between two days with head bobbing? | ☐ less than 7 days<br>☐ 1-2 weeks<br>☐ 2-4 weeks<br>☐ 1 month<br>☐ 2 months<br>☐ 3-4 months<br>☐ 5-6 months<br>☐ more<br>☐ I don't know<br>☐ my dog shows the head bobbing syndrome for less than 3 months |
| Have you ever recognized more than 1 tremor episode per day? If yes, how many and on how many days | ☐ yes ☐no<br>• If yes, how many episodes:<br>• If yes, on how many days: |
| | |
| Does your dog show any behavioral problems? (biting paws, hunting tail, aggresivity, …) | |
| Has epilepsy been diagnosed in your dog? | ☐ yes ☐no |
| Does your dog take anti-epileptic drugs? If yes, which ones? | ☐ yes ☐no<br>If yes, which ones |
| Does your dog take any drugs for treating the head bobbing? ? If yes, which ones? | ☐ yes ☐no<br>If yes, which ones |
| Are any other diseases diagnosed in your dog? If yes, which ones. | ☐ yes ☐no<br>If yes, which ones |
