# Supplementary material for: Imepitoin for treatment of idiopathic head tremor syndrome in dogs: A randomized, blinded, placebo‐controlled study
Source: J Vet Intern Med. 2020 Nov 7;34(6):2571–81. doi: 10.1111/jvim.15955 (PMC7694850; doi:10.1111/jvim.15955)
Supplement: Supplementary file 2 — Table S2 Patient and disease characteristics for design of matched pairs (format: PDF) [file JVIM-34-2571-s002.pdf]

Table S2: Patient and disease characteristics for design of matched pairs

Grade	Definition of characteristic	Subgroup
C1	Longest interval between two HT/HB episodes during baseline (T1)	S1: ≤ 14 days<br>S2: > 14 days
C2	Number of HT/HB days during 3-months-baseline period (Monthly HT/HB frequency)	S1: ≤ 6 days (≤2 HT/HB per month)<br>S2: > 6 days (≥2 HT/HB per month)
C3	Breeds	S1: Doberman Pinschers, Bulldogs, Boxers<br>S2: other breeds or mixed breeds
C4	Age of onset of HT/HB	S1: ≤ 1 year<br>S2: > 1 year
C5	Sex	S1: male<br>S2: female

Comparisons between the active and the control groups were made using matched pairs with one dog receiving imepitoin and its partner receiving placebo. In the process of matching, patient and HT/HB characteristics with potential impact on study outcome were assessed (C1 to C5). Characteristics were graded with C1 supposed to be the most and C5 being the less influencing characteristic. Matched pair partners were aimed to be similar in as many characteristics as possible in ascending order. Thus, if dogs differed in their characteristics, matching of characteristics focusing on frequency of the HT/HB episodes that were subsequently used for calculation of individual response to treatment (C1, C2) was preferred to matching of characteristics focusing on patient signalment (C3-C5).
