# Supplementary material for: Imepitoin for treatment of idiopathic head tremor syndrome in dogs: A randomized, blinded, placebo‐controlled study
Source: J Vet Intern Med. 2020 Nov 7;34(6):2571–81. doi: 10.1111/jvim.15955 (PMC7694850; doi:10.1111/jvim.15955)
Supplement: Supplementary file 3 — Table S3 Owner instruction for application of tablets and delayed application (format: PDF) [file JVIM-34-2571-s003.pdf]

Table S3: Owner instruction for application of tablets and delayed application (format: PDF)

Tablets application:

- We recommend to apply tablets 30 min before feeding (if this is not possible, the tablets can be applied with food, but application should be done in the same manner during the whole study phase).

- The tablets are administered 2 times daily every 12h, application time should not be changed during study phase

Instruction for delayed application:

- ≤ 2 hours delay: application is still considered „in time“. Apply tablet. The next tablet will be applyed as usually.

- 2-6 hours delay: application is considered „delayed application“. Documentation in tremor calender neceassary. Apply tablet. The next tablet will be applyed as usually.

- > 6 hours: application is considered „application omitted“. Documentation in tremor calender neceassary. Do not apply tablet. The next tablet will be applyed as usually

- If vomiting occurs immediately after tablet intake: Apply tablet again.
