# Supplementary material for: Imepitoin for treatment of idiopathic head tremor syndrome in dogs: A randomized, blinded, placebo‐controlled study
Source: J Vet Intern Med. 2020 Nov 7;34(6):2571–81. doi: 10.1111/jvim.15955 (PMC7694850; doi:10.1111/jvim.15955)
Supplement: Supplementary file 4 — Table S4 Single patient data: T1, T2, and T2/T1 in imepitoin and placebo group (format: PDF) [file JVIM-34-2571-s004.pdf]

Table S4: Single patient data: T1, T2 and T2/T1 in imepitoin and placebo group

Dog number	T1	T2	T2/T1
**Imepitoin Group**			
2	35	14	0,4
4	52	29	0,6
5	22	3	0,1
7	29	109	3,8
9	46	1	0,02
12	14	56	4,0
14	28	46	1,6
15	21	16	0,8
18	16	6	0,4
20	23	8	0,3
21	5	1	0,2
23	40	6	0,2
**Placebo Group**			
1	1	1	1,0
3	35	3	0,1
6	16	6	0,4
8	19	13	0,7
10	29	1	0,03
11	6	5	0,8
13	21	5	0,2
16	21	1	0,05
17	8	2	0,3
19	24	7	0,3
22	1	1	1,0
24	20	5	0,3

Single patient data in regard to T1, T2 and primary efficacy parameter T2/T1. Abbreviations: T1, the longest interval (days) between two head tremor days during the 3 months baseline period; T2: interval (days) between the second and the third head tremor day during study phase after completion of the titration phase; T2/T1, quotient T2 to T1, that evaluated the prolongation of the head tremor free period during study phase compared to baseline.

Responder are marked grey

Partial Responder are marked light grey
