# Supplementary material for: Imepitoin for treatment of idiopathic head tremor syndrome in dogs: A randomized, blinded, placebo‐controlled study
Source: J Vet Intern Med. 2020 Nov 7;34(6):2571–81. doi: 10.1111/jvim.15955 (PMC7694850; doi:10.1111/jvim.15955)
Supplement: Supplementary file 5 — Table S5 Trend of cluster days during study phase compared to baseline (format: PDF) [file JVIM-34-2571-s005.pdf]

Table S5: Trend of cluster days during study phase compared to baseline

	**Imepitoin, n = 12**<br>No of dogs (rate)	**Placebo, n = 12**<br>No dogs (rate)
Disappearance or decrease of clusters during study phase	5 (42%)	3 (25%)
Increase or new onset of clusters during study phase	4 (33%)	6 (50%)
No clusters during baseline and study phase	3 (25%)	3 (25%)
