# Supplementary material for: Imepitoin for treatment of idiopathic head tremor syndrome in dogs: A randomized, blinded, placebo‐controlled study
Source: J Vet Intern Med. 2020 Nov 7;34(6):2571–81. doi: 10.1111/jvim.15955 (PMC7694850; doi:10.1111/jvim.15955)
Supplement: Supplementary file 6 — Table S6 Single patient data: F1, F2 and delta F in imepitoin and placebo group (only for matched pairs treated for>6 weeks with active drug or placebo) (format: PDF) [file JVIM-34-2571-s006.pdf]

Table S6: Single patient data: F1, F2 and ΔF in imepitoin and placebo group (only for matched pairs treated for > 6 weeks with active drug or placebo), number of matched pairs = 7; number of dogs = 14

Dog number	F1	F2	ΔF
Imepitoin group			
4	5,0	5,0	0%
7	6,5	0,4	93%
12	3,1	0,0	100%
14	10,0	2,9	71%
15	2,5	1,4	45%
20	3,9	3,3	16%
23	1,2	2,0	-68%
Placebo Group			
3	2,8	3,5	-24%
8	2,2	1,1	49%
11	11,8	3,7	68%
13	7,5	1,7	78%
16	5,0	2,6	48%
19	3,2	4,8	-51%
24	2,2	1,4	37%

For dogs with a duration of study phase of ≥6 weeks ΔF was assessed as secondary efficacy parameter (conventional outcome parameter). ΔF was defined as %-reduction of mean number of monthly HT/HB days during study phase (F2) compared to baseline (F1): ΔF(%) = (1-F2/F1) x 100. Positive results indicated a decrease in HT/HB days, negative results indicated an increase in HT/HB days. Responders were defined as ≥ 50% decrease in HT/HB day frequency: ▢ Responder are marked grey.
Abbreviations: F1, mean number of head tremor days per month during the baseline period (monthly head tremor frequency); F2, mean number of head tremor days per month during the study phase; HT/HB, head tremor/head bobbing.
