# Supplementary material for: Imepitoin for treatment of idiopathic head tremor syndrome in dogs: A randomized, blinded, placebo‐controlled study
Source: J Vet Intern Med. 2020 Nov 7;34(6):2571–81. doi: 10.1111/jvim.15955 (PMC7694850; doi:10.1111/jvim.15955)
Supplement: Supplementary file 7 — Table S7 Different T2/T1 quotients for study patients with voluntary extension based on different T2 (format: PDF) [file JVIM-34-2571-s007.pdf]

Table S7: Different T2/T1 quotients for study patients with voluntary extension based on different T2

Dog No	**T2/T1 quotient based on different T2** (n = 15) *T2 representing interval between HT/HB episodes x-y in days*						Mean T2/T1
	1-2	2-3	3-4	4-5	5-6	6-7	
**Imepitoin Group**							
2	0,03	0,40	0,66	0,03	0,66	-	0,35
4	0,27	0,56	0,13	0,04	0,02	0,02	0,17
14	0,04	1,64	0,18	0,04	0,21	0,07	0,36
18	0,06	0,38	0,25	0,25	0,38	0,19	0,25
20	0,09	0,35	0,26	0,22	0,13	1,04	0,35
23	1,03	0,15	0,10	0,15	0,18	-	0,32
**Placebo Group**							
1	1,00	1,00	1,00	1,00	1,00	2,00	1,17
3	0,11	0,09	0,03	0,03	0,06	0,09	0,07
6	0,19	0,38	0,13	0,50	0,06	0,00	0,21
10	0,07	0,03	0,45	0,07	0,07	0,03	0,12
11	0,67	0,83	1,17	0,17	0,50	1,17	0,75
13	0,05	0,24	0,14	1,86	1,57	1,57	0,90
16	0,10	0,05	0,95	0,81	0,76	0,14	0,47
17	0,25	0,25	1,38	0,13	0,50	0,13	0,44
22	1,00	1,00	1,00	1,00	1,00	1,00	1,00

Different T2/T1 quotients for study patients based on different T2, representing different interepisodic intervals after the 2-week-titration phase for all dogs that continued blinded treatment voluntarily after their individual study endpoint was reached (voluntary extension: imepitoin 6; placebo 9). The interval between head tremor (HT/HB) episode 2-3 was used for efficacy evaluation in this study. The quotient T2/T1 between the 2nd and 3rd HT/HB episode appeared representative in comparison to other interepisodic intervals and the mean in most of the dogs. Dog 14 from the imepitoin group was characterized as a partial responder based on T2/T1 if the interval between the 2nd and 3rd HT/HB episode was used for calculation. Though later intervals did not support this finding. Dog 13 would have been characterized as a partial responder based on later intervals. This dog belonged to the placebo group.
Abbreviations: T1, the longest interval (days) between two head tremor days during the 3 months baseline period; T2: interval days) between the second and the third head tremor day during study phase after completion of the titration phase; T2/T1, quotient T2 to T1, that evaluated the prolongation of the head tremor free period during study phase compared to baseline.
